# The Blending of Poly(glycolic acid) with Polycaprolactone and Poly(l-lactide): Promising Combinations

**DOI:** 10.3390/polym13162780

**Published:** 2021-08-18

**Authors:** Luca Magazzini, Sara Grilli, Seif Eddine Fenni, Alessandro Donetti, Dario Cavallo, Orietta Monticelli

**Affiliations:** 1 Dipartimento di Chimica e Chimica Industriale, Università degli Studi di Genova, Via Dodecaneso 31, 16146 Genova, Italy; lucamaga95@gmail.com (L.M.); saragrilli.sg@libero.it (S.G.); seifeddinefenni@gmail.com (S.E.F.); 2 Natur-World S.p.A., Via Roma 8/2, 16121 Genova, Italy; a.donetti@natur-world.it

**Keywords:** PGA, PLA, PCL, blends

## Abstract

Poly(glycolic acid) (PGA) holds unique properties, including high gas barrier properties, high tensile strength, high resistance to common organic solvents, high heat distortion temperature, high stiffness, as well as fast biodegradability and compostability. Nevertheless, this polymer has not been exploited at a large scale due to its relatively high production cost. As such, the combination of PGA with other bioplastics on one hand could reduce the material final cost and on the other disclose new properties while maintaining its “green” features. With this in mind, in this work, PGA was combined with two of the most widely applied bioplastics, namely poly(l-lactide) (PLLA) and poycaprolactone (PCL), using the melt blending technique, which is an easily scalable method. FE-SEM measurements demonstrated the formation of PGA domains whose dimensions depended on the polymer matrix and which turned out to decrease by diminishing the PGA content in the mixture. Although there was scarce compatibility between the blend components, interestingly, PGA was found to affect both the thermal properties and the degradation behavior of the polymer matrices. In particular, concerning the latter property, the presence of PGA in the blends turned out to accelerate the hydrolysis process, particularly in the case of the PLLA-based systems.

## 1. Introduction

The concerns about the accumulating plastic waste pollution as well as the increasing environmental pressure on global warming have stimulated tremendous attention toward bioplastics [1,2,3]. Among them, poly(glycolic acid) (PGA) holds promising characteristics [4], such as excellent mechanical properties [5], high heat distortion temperature, as well as exceptionally high gas barrier properties [6,7,8]. Furthermore, the polymer chains, which have a similar chemical structure to poly(lactic acid) (PLA) but without the methyl side group, are tightly packed together, thus producing elevated thermal stability and high degree of crystallinity [4]. The latter unique feature, which affects the PGA properties, was studied in detail. Indeed, by using different characterization techniques, Sato et al. [9] demonstrated that the intermolecular interactions in PGA chains influence the achievable structure, which results in a high melting temperature. Moreover, it was reported that in the crystalline structure, the hydrogen bonds between the oxygen atoms of the ester group and the hydrogen atoms of CH_2_ are weak [10]. More recently, Báez et al. [11], comparing the crystallinity, the melting temperature (T_m_), and the enthalpy of fusion (ΔH_m_) of poly(l-lactide) (PLLA) and polycaprolactone (PCL) with those of PGA, observed that with a similar number average molecular weight, T_m_ was affected by the polarity of the structural unit of the ester, following the trend: PGA > PLLA > PCL. Clearly, the arrangement of crystalline domains, the intramolecular interactions as well as the conformation of molecular chains affect PGA’s mechanical properties. In particular, the structural regularity renders the polymer more rigid and stiff in comparison with other biopolymers [12]. Indeed, due to its highly crystalline structure, PGA has the strongest mechanical strength but the lowest fracture toughness in comparison to PLA, polylactic-co-glycolic acid (PLGA), and polyethylene terephthalate (PET) [4]. Another interesting feature of the polymer, which affects its extent of hydrolysis, is the relatively high hydrophilicity compared to other biopolymers, such as PLA and PCL [13,14,15,16]. As such, PGA hydrolytic degradation, whose rate is quite high, starts with the hydrolysis reaction with water and is followed by the random cleavage of the ester bonds in the polymer chain. Moreover, as occurs for other polyesters, the degradation rate is accelerated by the increase in the carboxylic end groups during the decomposition [17].

Although the aforementioned PGA characteristics make the polymer suitable for high-end applications, such as packaging, electronics, and automobile, so far, it has been mainly used in the biomedical field [18,19,20]. The main challenge of the application of PGA is related to its high production cost. As such, the development of novel manufacturing approaches, based for instance on the preparation of glycolic acid (GA) from renewable resources and industrial waste gases, could significantly decrease the production cost of PGA [21]. Moreover, the strategy of combining PGA with other biopolymers on one hand could diminish the material cost and on the other take advantage of the synergic properties of the blend components, while maintaining the environmental sustainability of the formulation. Although this approach could overcome some of the PGA drawbacks and enlarge its applicability, only very few studies reported on blends based on PLA and PCL, which are the polymers object of the present work.

Indeed, blends made by the mixing of PGA with PCL were mainly done in fiber/nanofiber form [22,23]. Spearman et al. [22] developed electrospun PCL/PGA nanofibers impregnated with double-stranded deoxyribonucleic acid wrapped single-walled carbon nanotubes and encapsulated by a PCL matrix. Pseudomonas lipase, which is used to catalyze the degradation, was found to promote the system degradation within four weeks. The degradation of fibers was also studied by Viera at al. [23], who compared the decomposition rate of neat and blend fibers based on PLA, PCL, PGA, and polydioxane (PDO) in various systems. Hydrolytic degradation was the fastest in the fibers composed by mixing PCL with PGA, whose degradation was more evident under basic conditions with respect to neutral ones.

Concerning PLA, the combination between the two polymers was mainly carried out by the copolymerization reaction, which allows obtaining PLGA, namely a copolymer of lactic acid and GA [24,25,26]. As for PCL, electrospun nanofiber blends based on PLA and PGA were developed, whose features turned out to be suitable for soft tissue engineering applications [27]. Moreover, a co-continuous phase morphology was obtained during the electrospinning process, and the subsequent extraction of PLA allowed producing porous fibers characterized by a very narrow pore size distribution [28]. Concerning films based on PGA/PLA blends, Ma et al. [29] applied the solution casting method, using the copolymer of PGA and PLA as compatibilizer. It was found that the crystallinity of PGA blended with the copolymer and with PLA decreased with increasing PLA content. In another study, the mechanical properties and degradation of PLA were improved by incorporating PGA fibers in PLA [30]. In this case, PGA fibers acted as reinforcement for PLA and increased the flexural strength and modulus.

It is worth underling that all the above examples are based on the dissolution of PGA by using expensive and highly impacting solvents, such as hexafluoroisopropanol, which is an approach that makes the material exploitation challenging. As such, with the aim of rendering the developed formulations more easily scalable, for the first time, melt blending was applied to develop blends based on PGA, PLA, and PCL. PGA was employed as a minority component in order to limit the costs of the final formulation. The prepared materials were studied in terms of thermal, mechanical, wettability, and degradation properties. Moreover, the influence of the blend composition on the morphology was evaluated by means of Field Emission Scanning Electron Microscopy (FE-SEM) measurements.

## 2. Materials and Methods

### 2.1. Materials

Poly(l-lactide) (PLLA), PLLA 1010 Synterra (average molecular weight 10^5^ g/mol, MFI = 12 g/10 min), was purchased from Purac (Amsterdam, The Netherland) in powder form. Polycaprolactone (PCL) was obtained from Perstorp (Malmö, Sweden) (CAPA 6500, Mn = 50.000 g/mol, MFI = 7 g/10 min), while poly(glycolic acid) (PGA), Vytal Resin (MFI = 20 g/10 min), was kindly received from Natur-World (Genoa, Italy).

### 2.2. Blend Preparation

Before accomplishing the blend preparation, in order to remove the moisture, the polymers were dried overnight in a vacuum oven at 60 °C for PLLA and PGA, while in the case of PCL, the temperature was set at 40 °C. Blends were prepared by using a Brabender Plastograph (Duisburg, Germany), mixing the components at 250 °C for 7 min and by applying a mixing speed of 100 rpm. The amount of material was constant in all the systems and corresponded approximately to a filling of 75% of chamber volume. In order to avoid the occurrence of degradation processes, some preliminary experiments allowed verifying the constancy of the torque in the chosen interval of time, as the decrease of the above parameter indicates a decrement of the polymer molecular weight. Moreover, also the neat polymers were treated using the same conditions applied in the blend preparation. The codes, which were assigned to the blends, indicate the ratio of the components (as an example, PCL70/PGA30 indicates a sample based on a ratio PCL/PGA of 70/30 composition).

### 2.3. Material Characterization

Thermal gravimetrical analysis (TGA) was performed with a Star^e^ System Mettler thermobalance (Milan, Italy). Samples, with weight of 5–8 mg, were heated from 35 to 800 °C at a heating rate of 10 °C/min, under a nitrogen flow of 80 mL/min.

Differential scanning calorimetry (DSC) experiments were performed with a TA Instruments Q20 (Milan, Italy), operating under flow of nitrogen. The samples, having a mass of about 5 mg, were firstly heated from room temperature to 250 °C; then, they were cooled down to 20 °C or −30 °C for PLLA-based and PCL-based blends, respectively. Finally, the samples were heated to 250 °C again. A scanning rate of 10 °C/min was used on both heating and cooling.

The degree of crystallinity (*X_c_*) was calculated by using Equation (1) and the enthalpies of fusion of 93 J·g^−1^, 139 J·g^−1^, and 191 J·g^−1^ for 100% crystalline PLLA [31], PCL [32], and PGA [33], respectively.

(1) $$X_{c}\left(\%\right)=\frac{\Delta H_{m}}{\Delta H_{0}}\times 100$$

where ∆*H_m_* is the measured heat of fusion and ∆*H*_0_ is the melting enthalpy of the 100% crystalline polymer.

DSC and TGA measurements were conducted in replicate.

Contact angle measurements were performed at room temperature with an Attension contact angle meter (Biolin Scientific, Gothenburg, Sweden) using pure distilled water and diiodomethane as probe liquids. The average static contact angles were obtained by measuring at least three droplets on each film specimen (two films for sample).

In order to obtain the surface energy, the following equation was applied:

(2) $$\frac{\gamma_{l}\left(1+\cos\theta \right)}{2\sqrt{\gamma_{l}^{d}}}=\sqrt{\gamma_{s}^{d}}+\sqrt{\gamma_{s}^{p}}\frac{\sqrt{\gamma_{l}^{p}}}{\sqrt{\gamma_{l}^{d}}}$$

where $\gamma_{s}^{p}$ is the sample surface energy polar component and $\gamma_{s}^{d}$ is the sample surface energy-dispersive component. For water: $\gamma_{l}$ = 72 mN/m; $\gamma_{l}^{p}$ = 51 mN/m; $\gamma_{l}^{d}$ = 21.8 mN/m. For diiodomethane: $\gamma_{l}$ = 50.8 mN/m; $\gamma_{l}^{p}$ = 1.3 mN/m; $\gamma_{l}^{d}$ = 49.5 mN/m.

A Zeiss Supra 40 VP field emission scanning electron microscope equipped with a backscattered electron detector (Oberkochen, Germany) was applied to examine the blend morphologies. The specimens were submerged in liquid nitrogen and fractured cryogenically. All samples were thinly sputter-coated with carbon using a Polaron E5100 sputter coater (Leica Microsystems, Wetzlar, Germany). Statistical analysis of PGA domains was performed by evaluating at least four micrographs at different magnifications for each sample.

To test the hydrolytic degradation, films of the neat polymers and of the blends based on a 70/30 ratio (previously dried overnight), having dimensions of 1 × 1 cm were prepared by a hot-pressing method using a Carver Hydraulic Lab Presses (Wabash, Indiana, USA). The specimens were immersed into 2 mL of distilled water at 50 °C. At different time intervals, the films were removed from water and vacuum dried. Three films were analyzed for each sample.

The (dry) sample weights were determined both before and after the immersion, and the extent of degradation was measured as percentage weight loss of the film as:

(3) $$\mathrm{Weight}\mathrm{loss}\left(\%\right)=\left[\frac{W_{dry0}-W_{dry}}{W_{dry0}}\right]\times 100$$

where *W_dry_*_0_ is the initial weight of the specimens and *W_dry_* is the dry weight of the specimens as measured after the treatment with water for a given time lapse.

## 3. Results

### 3.1. Blend Morphological Characterization

**Figure 1:**
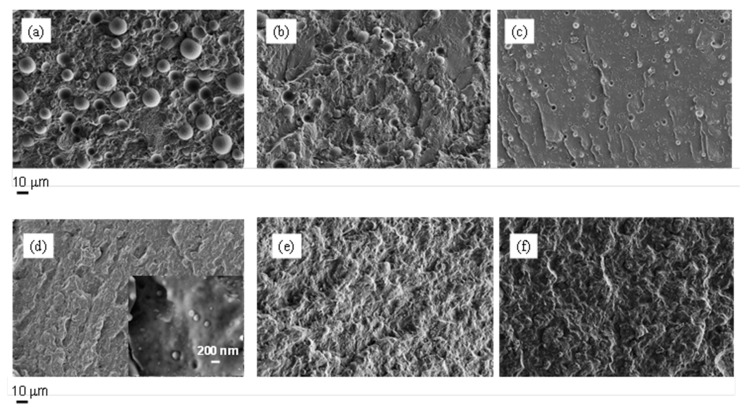
FE-SEM micrographs of: (**a**) PLLA70/PGA30, (**b**) PLLA80/PGA20, (**c**) PLLA90/PGA10, (**d**) PCL70/PGA30, (**e**) PCL80/PGA20, (**f**) PCL90/PGA10.

**Figure 2:**
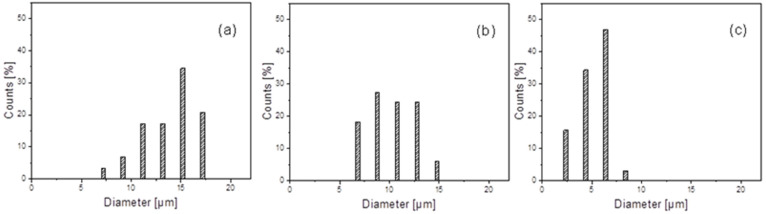
Histograms of the PGA domains diameter in the PLLA-based blends: (**a**) PLLA70/PGA30, (**b**) PLLA80/PGA20, and (**c**) PLLA90/PGA10.

The morphology of the prepared blends was studied by FE-SEM measurements. The micrographs obtained for the two investigated systems are shown in Figure 1. The PLLA/PGA blends show a typical sea-island morphology, where the spherical domains of PGA are dispersed in the PLLA matrix, thus demonstrating, at least for the analyzed compositions, the immiscibility between the two phases. Considering the dimensions of the PGA domains in the system PLLA/PGA (Figure 2), it is worth underling that their average diameter tends to decrease significantly, reducing the amount of the polymer in the blend, passing from 14 μm in the case of PLLA70/PGA30 sample to 5 μm for the blend PLLA90/PGA10.

On the other hand, in the case of PCL-based blends, the morphological analysis does not reveal the presence of measurable PGA domains. Only for the PCL70/PGA30 sample, considering the measurements carried out at high magnification (see insert of Figure 1d), PGA droplets (characterized by an average diameter of ca. 200 nm and which resulted partially attached to the polymer matrix) are clearly visible.

In order to explain the different behavior of the two investigated polymers, it is worth underling that the morphology of blends is affected by the viscosity and phase elasticity of the components as well as by the shear rate and blend composition [34]. Taking into account that the systems were prepared by applying the same conditions, it is possible to infer that the specific features of the polymers influence the final blend morphology.

### 3.2. Wettability Tests

The wettability of the films based on PLLA, PGA, and PCL was compared with that of the corresponding blends. In particular, to more easily verify the specific effect of PGA on the properties of the systems, blends containing the highest amount of the polymer, namely those with a 70/30 ratio, were studied. In order to evaluate the surface energy of the various systems, the contact angle of water and diiodomethane was measured (Table 1). The water and diiomethane contact angle of the neat polymer films are in good agreement with those reported in the literature [35,36,37]. Indeed, the presence of PGA in the blend determines a significant decrease in the water contact angle due to the higher wettability of the above polymer compared to PLLA and PCL. Although the contact angle values are similar for the two systems, interestingly, the polar component of the PLLA-based systems is higher than the PCL/PGA blends. Clearly, this property might affect the behavior of the systems, especially in terms of degradability.

### 3.3. Study of the Hydrolytic Degradation

**Figure 3:**
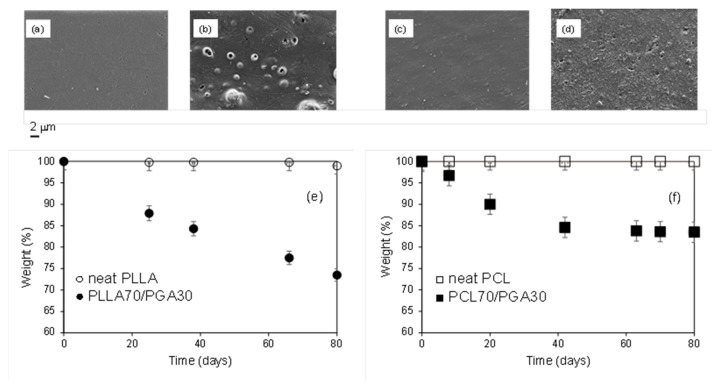
FE-SEM micrographs of treated films: (**a**) neat PLLA, (**b**) PLLA70/PGA30, (**c**) neat PCL, and (**d**) PCL70/PGA30. Weight loss percentage as a function of time of (**e**) films based on PLLA (○ neat PLLA, ● PLLA70/PGA30) and (**f**) films based on PCL (□ neat PCL and ■ PCL70/PGA30).

The hydrolytic degradation of the prepared blends was studied by monitoring the weight loss over time for specimens placed in water. As previously reported, blends with a 70/30 ratio were considered, and with the aim of speeding up the process, the measurements were carried out at 50 °C. Furthermore, the morphology of the surfaces of the films, which underwent a hydrolysis treatment for 80 days, was analyzed by FE-SEM measurements. In order to verify the effect of the presence of PGA, which was dispersed in the two polymer matrices, the behavior of the neat PLLA and PCL films was also analyzed. Indeed, Figure 3 shows the FE-SEM micrographs of the neat PLLA (Figure 3a) and PCL (Figure 3c) treated films and those of the treated blends PLLA70/PGA30 (Figure 3b) and PCL70/PGA30 (Figure 3d), together with the percentage weight loss versus time.

In the analyzed time interval (80 days), the weight loss of the neat PLLA and PCL-based films is practically negligible, and the films’ surface appeared homogeneous (Figure 3a,c). It is worth underling that in all the performed experiments, the deviation of the weight loss was within 2%. Indeed, the slow degradation rate of both the polymers, even under very accentuated hydrolysis conditions, that is directly in water and at high temperature, cannot satisfy a wide range of application specific requirements [38,39]. In this regard, it is worth underling that the need to accelerate the degradation process is essential not only for bio-medical applications [40], as an example when the polymer matrices are used as drug carriers, but also in other fields, such as packaging. Considering the trends shown in Figure 3, it is evident that the presence of PGA in the blends significantly modifies the degradation, accelerating the process. Nevertheless, the kinetic of the system decomposition turned out to depend on the polymer matrix, being faster in the case of PLLA-based blends. It is worth noticing that the weight loss kinetic is almost the same for both the blends in the first 40 days, but afterwards, it slows down for those based on PCL.

In order to clarify the above phenomenon, it is necessary to consider the degradation mechanism of PGA as well as the specific properties in terms of morphology, crystallinity, and wettability of the prepared blends. Concerning the former aspect, it was demonstrated that the degradation of PGA occurs in four stages, which are driven both by the water diffusion and by the hydrolysis rate [13,14,15,16,41,42]. Indeed, in the first step, the water diffuses into the polymer, reaching its maximum concentration within a few hours, while in stage two, the diffusion of water becomes slower and the polymer molecular weight decreases steadily as hydrolysis reaction begins. The decrement of molecular weight as well as the plasticizing effect of water lead to an increase of chain mobility in the amorphous phase, which promotes a secondary crystallization. In stage three, the oligomers, formed in stage two, start to diffuse from the polymer surface into the solution. As more spaces are created in the polymer, more water can diffuse through the bulk of the polymer, which is a phenomenon that accelerates the reaction and creates sharp erosion fronts throughout the sample. Indeed, it is possible to hypothesize that the lower crystallinity as well as the higher polar component of the PLLA-based systems favor the water diffusion, resulting in a more constant degradation rate over time than that of the PCL/PGA blends. Furthermore, in the PLLA-based blends, as it appears from the micrograph of the treated sample (Figure 3b), the degradation seems confined in the micrometer-size PGA domains. Conversely, the finer PGA dispersion in the PCL/PGA blends promotes a more distributed erosion (Figure 3d). Summarizing, the effective role of PGA in tuning the degradation of biopolymers was verified by studying systems easily prepared by applying the scalable melt blending technique.

### 3.4. Study of the Thermal Properties

**Figure 4:**
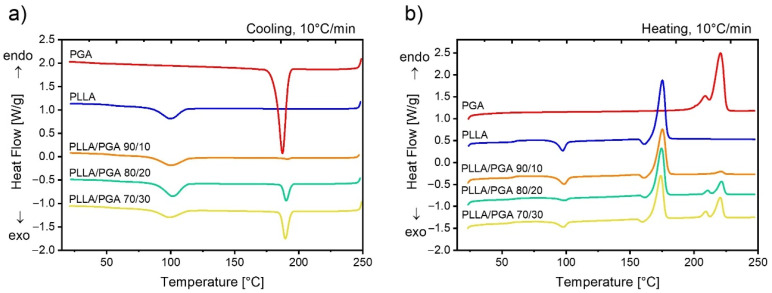
DSC cooling (**a**) and heating (**b**) scans for the neat polymers and PLLA/PGA blends of indicated compositions.

Figure 4 displays the characteristic DSC scans upon cooling and heating PLLA, PGA, and their blends. The thermal properties for all the samples are collected in Table S1 of the Supporting Information. The different crystallizability of the two neat polymers can be noticed. In fact, PGA crystallizes with a large and relatively narrow peak at around 190 °C, while the crystallization of PLLA occurs only for a minor fraction in a broad interval centered at about 100 °C. Moreover, PLLA crystallization is incomplete on cooling, as it can be judged from the cold crystallization event on subsequent heating above the glass transition, again at 100 °C. The initial crystallinity of PLLA, before heating, is ca. 40%. The high crystallinity of PGA (*X_c_* of ca. 45%) prevents the observation of a distinct glass transition, which should anyhow be present at 35–40 °C [33]. A double melting peak is observed for PGA, and it is tentatively ascribed to melting–recrystallization phenomena, which is typical of many polyesters (see [43] and reference therein).

The DSC scans of the PLLA/PGA blends simply show the thermal features of both polymers to an extent representative of the relative content. In particular, it should be noticed that neither the crystallization temperatures nor the melting temperatures of the components are affected by the blend composition. This indicates an immiscibility between the two components, which is in line with morphological observation examined via FE-SEM (Figure 1).

**Figure 5:**
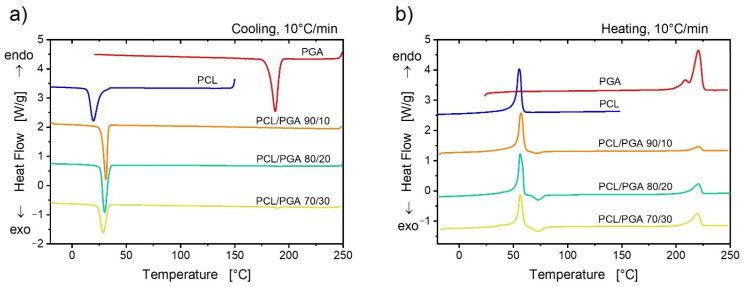
DSC cooling (**a**) and heating (**b**) scans for the neat polymers and PCL/PGA blends of indicated compositions.

Figure 5 shows the differential scanning calorimetry results of PCL/PGA blends, along with those of the respective neat polymers. PGA and PCL are widely separated for what concerns their crystallization and melting temperatures. In fact, a difference of more than 150 °C can be observed between the respective transitions. Moreover, the crystallinity of neat PCL is ca. 45%. Contrary to what has been observed for the case of PLLA/PGA blends, a noteworthy effect of the blending process on the crystallization/melting of the two phases can be observed. Upon cooling, it can be seen that the crystallization of the PGA phase at high temperature is practically suppressed, while the crystallization temperature of PCL is enhanced with respect to that of the neat polymer. The first effect is expected, given the extremely limited size of the PGA domains, and it is almost not resolvable with FE-SEM. As a consequence, a very small number of nucleating impurities is present in the PGA domains, leading to vanishing crystallization kinetics [44]. As a matter of fact, PGA can only crystallize upon the subsequent heating, as evidenced by the cold-crystallization peak observed above the melting temperature of PCL, at around 70 °C. On the other hand, the increase of the crystallization temperature of PCL can be ascribed to a nucleating effect of PGA domains themselves, or to an effective transfer of nucleating impurities from the PGA to the matrix polymer.

However, despite the observed kinetics effect on crystallization, the melting temperatures of the two phases are unaffected by the blending process. In particular, no evident melting point depression could be detected, indicating once more a thermodynamic immiscibility between the polymers, despite the morphology indicating a quite good compatibility (Figure 1).

**Figure 6:**
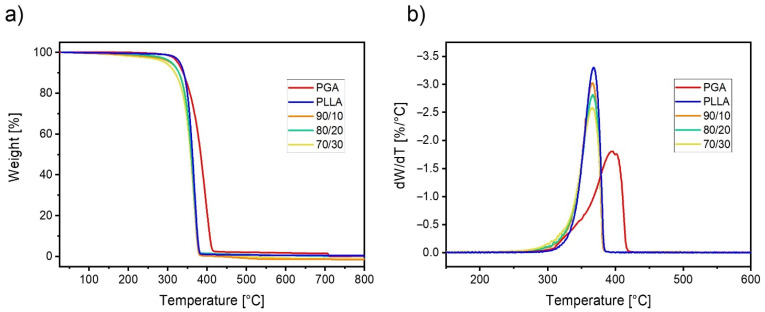
Mass loss versus temperature (**a**) and its derivative (**b**) for neat PLLA, PGA, and their blends at the indicated compositions.

The results of thermogravimetric analysis for the PLLA/PGA blends and neat polymers are reported in Figure 6. It can be seen that PGA exhibits a higher thermal stability with respect to PLLA, displaying in particular a similar degradation onset temperature but a ca. 30 °C higher temperature of maximum degradation rate, T_max_ (Figure 6b). The thermal stability of the blends is not intermediate between the two neat polymers but is instead similar to that of neat PLLA, with a slightly lower onset temperature but identical T_max_. These results confirm that during the melt blending process, the molecular features of the PLLA matrix did not undergo substantial changes that could decrease the resistance to temperature.

**Figure 7:**
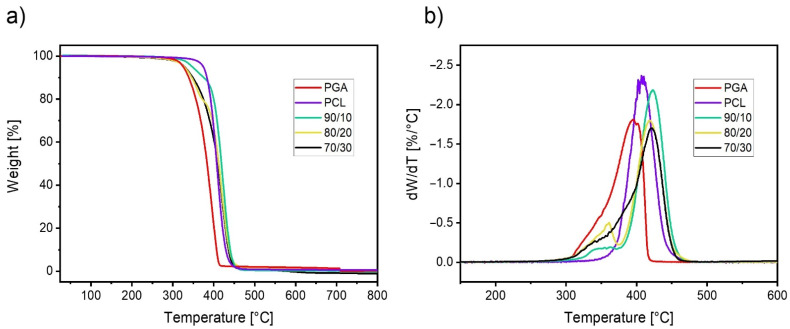
Mass loss versus temperature (**a**) and its derivative (**b**) for neat PCL, PGA, and their blends at the indicated compositions.

Thermogravimetric data for the system PCL/PGA are shown in Figure 7. PCL possesses a higher thermal stability with respect to PGA. In particular, the onset of PCL degradation is located approximately 50 °C above that of PGA. In addition, the maximum rate of degradation is shifted to higher temperatures (Figure 7b). The behavior of the blends is somehow intermediate between the two neat polymers. In fact, there is a fraction of the material, increasing with the content of PGA, which degrades earlier than neat PCL, while the majority of the blend thermally degrades in a temperature interval superposed or similar to the one of PCL. As such, the thermal stability of the blend is only partially compromised with respect to the more resistant polymer.

## 4. Conclusions

In this work, with the aim of verifying the possibility of extending the applicability of PGA, a promising biopolymer suffering from a still high production cost, formulations were developed by combining it, as a minor component, with PLLA and PCL, which are two of the most extensively exploited biopolymers. In order to make the study closer to an industrial development, unlike some works reported in the literature where blends were prepared starting from solutions, the melt blending technique, which is an easily scalable method, was applied. The morphology of the prepared blends strongly depended on the polymer matrix: the PGA domains, which were not very adherent to both the polymeric matrices, turned out to be extremely smaller in the case of the PCL-based systems compared to the PLLA/PGA blends. Moreover, for both the investigated systems, the wettability increased considerably with the addition of PGA. This caused an interesting increment in the blends degradation rate with respect to that of the polymeric matrices, which is an extremely important feature for a practical application of the developed systems. Furthermore, this phenomenon, which is related to the specific characteristics of the blend as well as of the polymeric matrix such as the crystallinity, the size of PGA domains, and the polar component of the surface energy, was more relevant in the case of blends based on PLLA, demonstrating the possibility of tuning the degradation by properly choosing the system.

For what concerns the crystallization behavior, PLLA and PGA crystallized and melted separately in their relatively coarse blends, at temperatures typical of the pure component, without showing any meaningful interaction. On the other hand, the blending of PCL with PGA caused pronounced mutual effects on the crystallization of the components. In particular, the PCL crystallization temperature was increased, while PGA crystallization in the extremely small domains was hindered, as it occurred only in cold crystallization upon second heating. Thermogravimetric analysis of the prepared blends revealed that the thermal stability of the matrix was not negatively affected by the presence of a low content of PGA, both for the cases of PLLA, which degraded before PGA, and of PCL, with higher thermal stability compared to PGA.

In conclusion, the feasibility of a melt blending approach for the preparation of biobased blends containing PGA was demonstrated. The obtained mixtures exhibited interesting characteristics, especially regarding their hydrolytic degradation behavior, which is tunable with respect to the matrix polymers. This preliminary work will serve as a basis to further explore the characteristics, especially the mechanical properties, and the potential application of these novel materials exploiting the environmentally friendly PGA.

## Figures and Tables

**Table 1:** Water, diiodomethane contact angles, and surface energies of the neat polymers and of the PLLA70/PGA30 and PCL70/PGA30 blends.

Sample Code	Water	Diiodomethane	γ_s_	$\gamma_{s}^{p}$	$\gamma_{s}^{d}$
	Contact Angle	Contact Angle			
	[°]	[°C]	[mN/m]	[mN/m]	[mN/m]
PLLA	80 ± 1.2	44 ± 2.8	41.9	8.8	33.1
PCL	75 ± 1.3	26 ± 2.5	46.8	4.6	42.2
PGA	50 ± 1.3	20 ± 2.1	58.1	18.2	39.9
PLLA70/PGA30	63 ± 3.8	41 ± 2.2	47.1	13.5	33.6
PCL70/PGA30	64 ± 1.4	22 ± 2.4	49.7	7.3	42.4

γ_s_ = surface energy, $\gamma_{s}^{p}$ = polar component of surface energy, and $\gamma_{s}^{d}$ = dispersive component of surface energy.

## Data Availability

The data that support the findings of this study are available from the corresponding author upon reasonable request.

## Supplementary Materials

The following are available online at https://www.mdpi.com/article/10.3390/polym13162780/s1, Table S1: DSC analysis of the neat polymers and polymer blends, Table S2: TGA analysis of the neat polymers and polymer blends.
