# Supplementary material for: The Blending of Poly(glycolic acid) with Polycaprolactone and Poly(l-lactide): Promising Combinations
Source: Polymers (Basel). 2021 Aug 18;13(16):2780. doi: 10.3390/polym13162780 (PMC8400216; doi:10.3390/polym13162780)
Supplement: Supplementary file 1 [file polymers-13-02780-s001.zip › polymers-1338256-supplementary.pdf]

Table S1. DSC analysis of the neat polymers and polymer blends. The values of melting temperature (T_m), melting enthalpy (ΔH_m), crystallization temperature (T_c), crystallization enthalpy (ΔH_c), cold-crystallization temperature (ΔH_{cc}), cold-crystallization temperature (T_{cc}), glass transition temperature (T_g) and crystallinity (X_c) are reported. The values of enthlpy and crystallinity of the various components are normalized by the relative polymer content.

System	T_m, °C	ΔH_m, J/g	T_c, °C	ΔH_c, J/g	T_{cc}, °C	ΔH_{cc}, J/g	T_g, °C	X_c, %
PLLA	175	54	99	-32	97	-17	60	40
PGA	221	83	187	-86				45
PCL	55	63	20	-58				45
PLLA/PGA 90/10								
PLLA	175	49	99	-26	98	-15	59	37
PGA	222	32	190	-13				17
PLLA/PGA 80/20								
PLLA	175	51	100	-36	99	-10	60	44
PGA	221	65	190	-62				34
PLLA/PGA 70/30								
PLLA	174	54	98	-28	98	-18	58	39
PGA	220	66	190	-66				35
PCL/PGA 90/10								
PCL	57	59	31	-56				42
PGA	221	65			72	-42	40	12
PCL/PGA 80/20								
PCL	56	73	30	-64				52
PGA	220	90	192	-4	73	-40	39	26
PCL/PGA 70/30								
PCL	56	57	29	-53				41
PGA	220	83	189	-12	73	-37	37	24

Table S2. TGA analysis of the neat polymers and polymer blends. The characteristic temperature to obtain 1, 5 and 10% mass loss, i.e., $T_{1\%}$, $T_{5\%}$ and $T_{10\%}$, are reported.

System	$T_{1\%}$, °C	$T_{5\%}$, °C	$T_{10\%}$, °C
PLLA	240	291	305
PGA	292	330	344
PCL	333	380	389
PLLA/PGA 90/10	173	312	334
PLLA/PGA 80/20	214	314	333
PLLA/PGA 70/30	178	290	310
PCL/PGA 90/10	307	353	381
PCL/PGA 80/20	270	332	351
PCL/PGA 70/30	258	333	354
